# ASCO 2016: highlights in breast cancer

**DOI:** 10.1007/s12254-016-0300-6

**Published:** 2016-11-30

**Authors:** Rupert Bartsch, Elisabeth Bergen

**Affiliations:** 1 Comprehensive Cancer Center Vienna, Vienna, Austria; 2 Department of Medicine 1, Clinical Division of Oncology, Medical University of Vienna, Waehringer Guertel 18–20, 1090 Vienna, Austria

**Keywords:** ASCO Annual Meeting 2016, Breast cancer, Highlights, Review

## Abstract

At the 2016 ASCO Annual Meeting, several pertinent studies in the field of breast cancer were presented. MA17.R was the first randomized phase III trial to evaluate the prolongation of adjuvant aromatase-inhibitor (AI) therapy from 5 to 10 years; while a significant reduction of disease-free survival events was observed in the extended treatment group, the absolute difference was relatively small and longer endocrine therapy resulted in a higher fracture rate. A combined analysis of three North American trials emphasized the superiority of anthracycline containing adjuvant chemotherapy regimens compared with docetaxel/cyclophosphamide (TC), while the PANTHER trial investigated dose-dense tailored adjuvant treatment. In metastatic breast cancer, the main interest was on cyclin-dependent kinase (CDK) 4/6 inhibitors. In PALOMA-2, the addition of palbociclib to letrozole prolonged progression-free survival (PFS) from 14.5 to 24.8 months resulting in the longest PFS data ever reported in the first-line setting. A subgroup analysis of premenopausal patients accrued to PALOMA-3 indicated that in this patient subset, ovarian function suppression plus fulvestrant and palbociclib yielded results comparable to the postmenopausal population. *ESR1* mutations were another focus of interest as these activating mutations in the gene coding for the estrogen receptor alpha apparently evolve under the selection pressure of AI therapy.

## Extended adjuvant therapy

MA.17R was the first study evaluating the extension of aromatase inhibitor (AI) therapy to 10 years [1]. Of note, in hormone-receptor (HR)-positive breast cancer (BC), a prolonged albeit low recurrence risk has been reported with the greatest risk observed in large, node-positive and G3 tumors [2].

Patients were randomly assigned to receive 5 years of letrozole or placebo after an initial 5 years of letrozole either upfront or after 2–5 years of tamoxifen. At a median follow-up of 6.3 years, extended AI therapy significantly improved 5‑year disease-free-survival (DFS) rates from 91 to 95% (HR = 0.61, *p* = 0.01) while no overall survival (OS) difference was reported. Furthermore, a significant increase of fracture rates was observed in the letrozole group. Regarding quality-of-life (QoL), a substudy indicated that prolongation of endocrine therapy was not associated with a significant overall QoL reduction but patients in the letrozole group had significantly higher rates of vasomotor symptoms and sexual dysfunction [3]. In summary, these data indicate that potential benefits of extended endocrine therapy must be carefully balanced with treatment tolerability and fracture risk.

## Adjuvant chemotherapy

Blum et al. presented data from a combined analysis of the NSABP B‑46I, NSABP B‑49, and USOR 06-090 studies (ABC trials) comparing six cycles of TC (docetaxel/cyclophosphamide) with standard regimens consisting of four cycles of taxane-based chemotherapy followed by four cycles of AC (doxorubicin/cyclophosphamide). The 3‑year invasive DFS was 91.7% in the TC group compared with 92.4% in patients with conventional therapy (HR = 1.202, *p* = 0.026). Therefore, TC was not non-inferior to conventional therapy and performed even significantly worse; as expected, a higher secondary leukemia rate was reported in the anthracycline group. Still, these data suggest that in the absence of contraindications, anthracycline-containing regimens remain the standard of care [4]. PANTHER, also known as ABCSG-25, was an open-label phase III study comparing dose-dense tailored (ddt) with conventional adjuvant chemotherapy [5]. Patients with node-positive or high-risk node-negative BC were randomized to three cycles of FEC100 followed by three cycles of docetaxel 100 mg/m² or EC (epirubicin/cyclophosphamide) × 4 followed by docetaxel × 4 given once every 2 weeks (dose-dense) and dose adapted to the leukocyte nadir. The main hypothesis tested in this study was the assumption that chemotherapy dose adaption by hematological toxicity may be more appropriate than chemotherapy dosing by body surface area.

At a median follow-up of 5.3 years, fewer BC recurrence-free survival (BCRFS) events were recorded in the ddt arm (HR = 0.79, 95% CI = 0.62–1.02, *p* = 0.064) while QoL, on the other hand, was significantly worse [6]. Of note, while ddt therapy was numerically superior, these patients received a greater overall number of chemotherapy cycles, a dose-dense regimen, and a regimen tailored by leukocyte nadir, making interpretation of the results difficult.

## Neoadjuvant therapy

The phase III ETNA trial asked whether nab-paclitaxel was superior to conventional paclitaxel when used as part of preoperative chemotherapy [7]. Patients received four cycles of conventional paclitaxel (90 mg/m²; days 1 + 8 + 15 of a 4-week cycle) or nab-paclitaxel (125 mg/m²) followed by four cycles of anthracycline-based therapy. In this study, relatively low pCR rates of 18.1% (conventional paclitaxel) and 22.5% (nab-paclitaxel) were observed and no significant difference between the two groups was recorded.

These data contradict the results of the GeparSepto trial where nab-paclitaxel improved pCR rates significantly [8]; of note, the regimen chosen in ETNA may have caused this difference. While GeparSepto used a standard regimen of paclitaxel or nab-paclitaxel weekly × 12, a regimen similar to the metastatic setting was used in ETNA resulting in a lower taxane dose density.

A British group reported DFS and OS outcomes of the phase III ARTemis trial. The primary endpoint analysis had already been published and a significantly higher pCR rate was observed when bevacizumab was added to neoadjuvant chemotherapy (22% vs. 17%, *p* = 0.03) [9]. As shown at this year’s ASCO Annual Meeting, this difference did not translate into superior outcomes in terms of DFS and OS [10]. In patients receiving chemotherapy alone, pCR predicted a favorable long-term outcome (pCR 97% vs. non-pCR 76%, *p* = 0.0006) while no such correlation was seen in the bevacizumab group (pCR 84% vs. non-pCR 74%, *p* = 0.19). These data, at first glance, contradict the paradigm that pCR is a predictor of favorable long-term outcome in high-risk BC subtypes. With bevacizumab-containing treatment, however, this correlation seems to be less clear as bevacizumab does not have any effect on micrometastases as indicated by the negative adjuvant studies.

In the neoadjuvant treatment of HER2-positive BC, the KRISTINE trial compared six cycles of T‑DM1 plus pertuzumab with docetaxel, carboplatin, trastuzumab, and pertuzumab (TCPH) [11]. Patients receiving TCPH had a significantly higher pCR rate (55.7% vs. 44.4%, *p* = 0.0155) and this effect was even more pronounced in non-luminal HER2-positive tumors. Toxicity rates, on the other hand, were higher in the TCPH arm as well.

While a lower pCR rate may be expected with T‑DM1 compared with conventional therapy, pCR rates in the KRISTINE trial compare well to the results from the luminal B/HER2-positive cohort of the ADAPT trials where T‑DM1 alone yielded a pCR rate of 41% [12]. This indicates that – similar to the metastatic setting – T‑DM1 has considerable activity that cannot be further improved by the combination of pertuzumab [13].

## Metastatic breast cancer

The main interest in the field of metastatic BC was in cyclin-dependent kinase (CDK) 4/6 inhibitors, a class of orally available drugs blocking cell-cycle progression.

The phase III PALOMA-2 trial allocated 666 patients with HR-positive/HER2-negative disease to letrozole plus the CDK 4/6 inhibitor palbociclib or letrozole plus placebo [14]. Patients with resistance to AIs (defined as progression on or within 12 months of adjuvant AI therapy) were excluded. Addition of palbociclib to endocrine therapy improved progression-free survival (PFS) from 14.5 to 24.8 months (HR = 0.58, 95% CI = 0.46–0.72, *p* < 0.000001), resulting in the longest PFS data ever reported in the first-line setting. Treatment was well tolerated with neutropenia being the most common side effect (79.5% vs. 6.3%); febrile neutropenia, however, was rare (2.5% in the palbociclib group). While it is currently not clear whether patients progressing on palbociclib will respond to further endocrine therapy, it appears that this relevant prolongation of DFS will ultimately define a novel first-line treatment standard.

In contrast to PALOMA-2, pretreated women were included in the PALOMA-3 trial and randomized to fulvestrant plus/minus palbociclib. The inclusion of premenopausal women who received additional ovarian function suppression was allowed; in this subset, the addition of palbociclib increased PFS from 5.6 to 9.5 months, suggesting that treatment activity in premenopausal subjects was comparable to the general study population [15].

A correlative study of PALOMA-3 investigated the prognostic and predictive role of activating *ESR1* mutations, a gene coding for the estrogen receptor alpha [16]. *ESR1* mutations were present in 26.8% of all samples and were only recorded in patients with prior AI exposure, leading to the hypothesis that *ESR1* mutations develop under the selection pressure of AI therapy. In PALOMA-3, the presence of the *ESR1* mutation was predictive of shorter PFS but the activity of palbociclib plus fulvestrant was independent of mutation status.

Finally, the development of immunotherapy was clearly delayed in BC. Current studies are focusing on the investigation of immune-checkpoint modulators in TN disease. In a phase Ib study, patients with metastatic TN BC received a combination of nab-paclitaxel plus atezolizumab, a humanized monoclonal antibody targeting PD-L1 [17]. The safety cohort of this study consisted of 32 patients, while 24 subjects were evaluable for response. The grade 3/4 neutropenia rate was 41%, but no dose-limiting toxicity was recorded. Of note, acitivity was promising with high response rates across all treatment lines independently of PD-L1 expression. Owing to these favorable outcomes, a corresponding phase III trial (IMPASSION; NCT02425891) is currently ongoing.

## Conclusion

In summary, the results from several relevant trials were presented at the 2016 ASCO Annual Meeting; of note, the results of the PALOMA-2 trial may prove practice changing.

### Take-home message

MA17.R indicated that the extension of AI therapy to 10 years was associated with a significant reduction of DFS events, but this difference was modest in absolute numbers and fracture rates were significantly increased. A combined analysis showed that conventional anthracycline-containing regimens were superior to six cycles of the anthracycline-free TC regimen. In the PANTHER study, ddt chemotherapy was compared with conventionally dosed FEC-Doc. While a numerical improvement was observed in the ddt arm, this difference did not reach statistical significance and QoL was significantly lower in the experimental group.

In metastatic BC, the PALOMA-2 trial showed that the addition of palbociclib to first-line letrozole resulted in a clinically relevant prolongation of PFS from 14.5 to 24.8 months. Besides CDK 4/6 inhibitors, the prognostic and predictive role of *ESR1* mutations was another focus of interest and currently available data suggest that *ESR1* mutations may evolve under the selection pressure of AI therapy. Finally, the combination of chemotherapy and PD-L1 inhibitors holds promise in metastatic TN disease.
